# Supplementary figures and images for: Novel monoclonal antibodies against house dust mite allergen Der p 21 and their application to analyze allergen extracts
Source: PeerJ. 2024 Apr 18;12:e17233. doi: 10.7717/peerj.17233 (PMC11032652; doi:10.7717/peerj.17233)

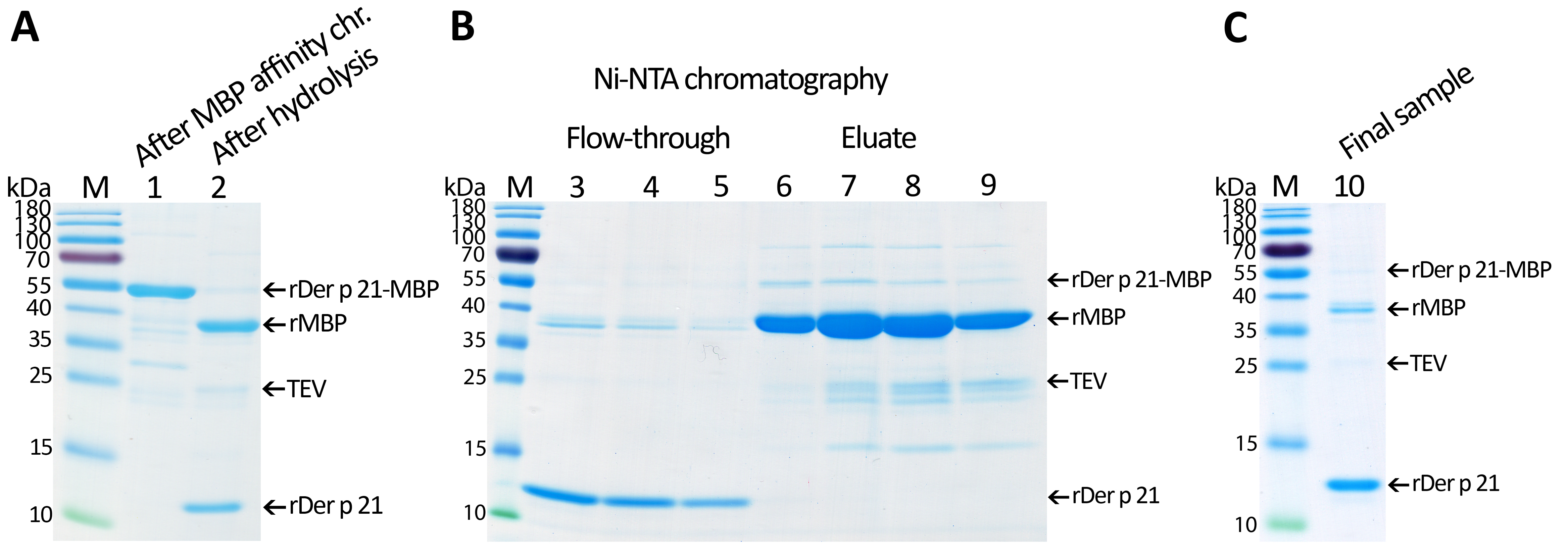

Supplement: Supplemental Information 1 — (A) rMBP-Der p 21 after the MBP affinity chromatography and after the hydrolysis reaction. (B) Purification of rDer p 21 by immobilized metal affinity chromatography. (C) Final sample of purified rDer p 21. [file peerj-12-17233-s001.png]

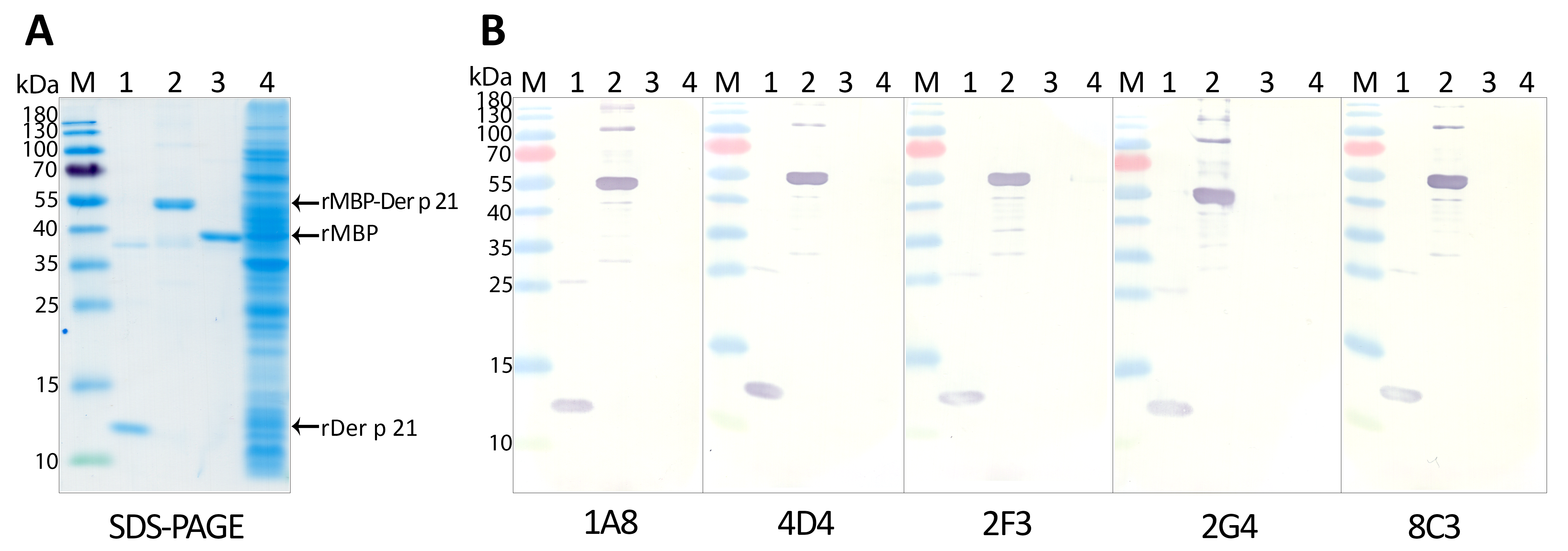

Supplement: Supplemental Information 2 — (A) SDS-PAGE and (B) Western blot. Lane 1 - rDer p 21; lane 2 - rMBP-Der p 21; lane 3 - rMBP; lane 4 - E. coli BL21 Star™ (DE3) lysate. [file peerj-12-17233-s002.png]

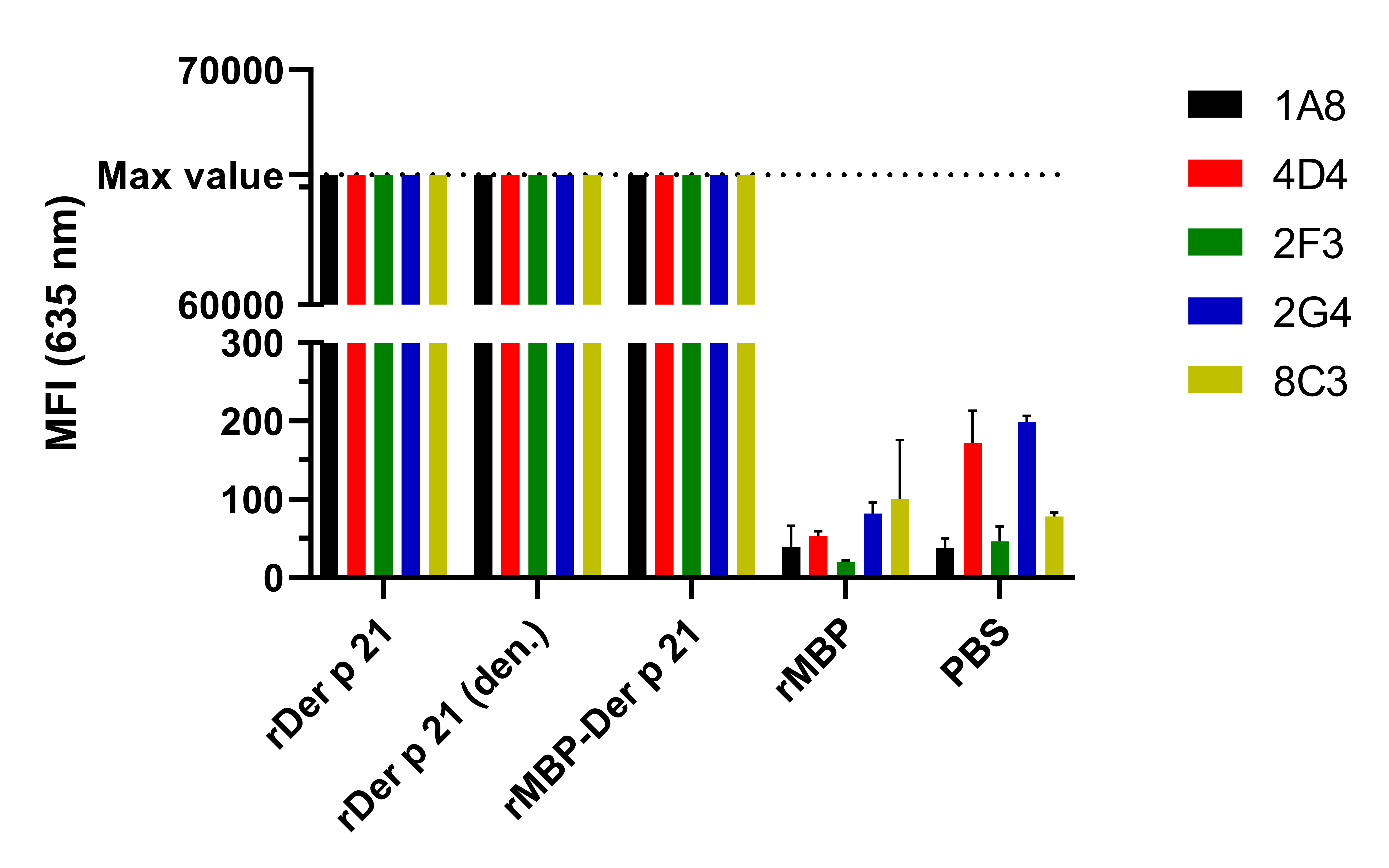

Supplement: Supplemental Information 3 — Max detectable intensity value is 65,535. n = 3, mean ± SD. [file peerj-12-17233-s003.png]

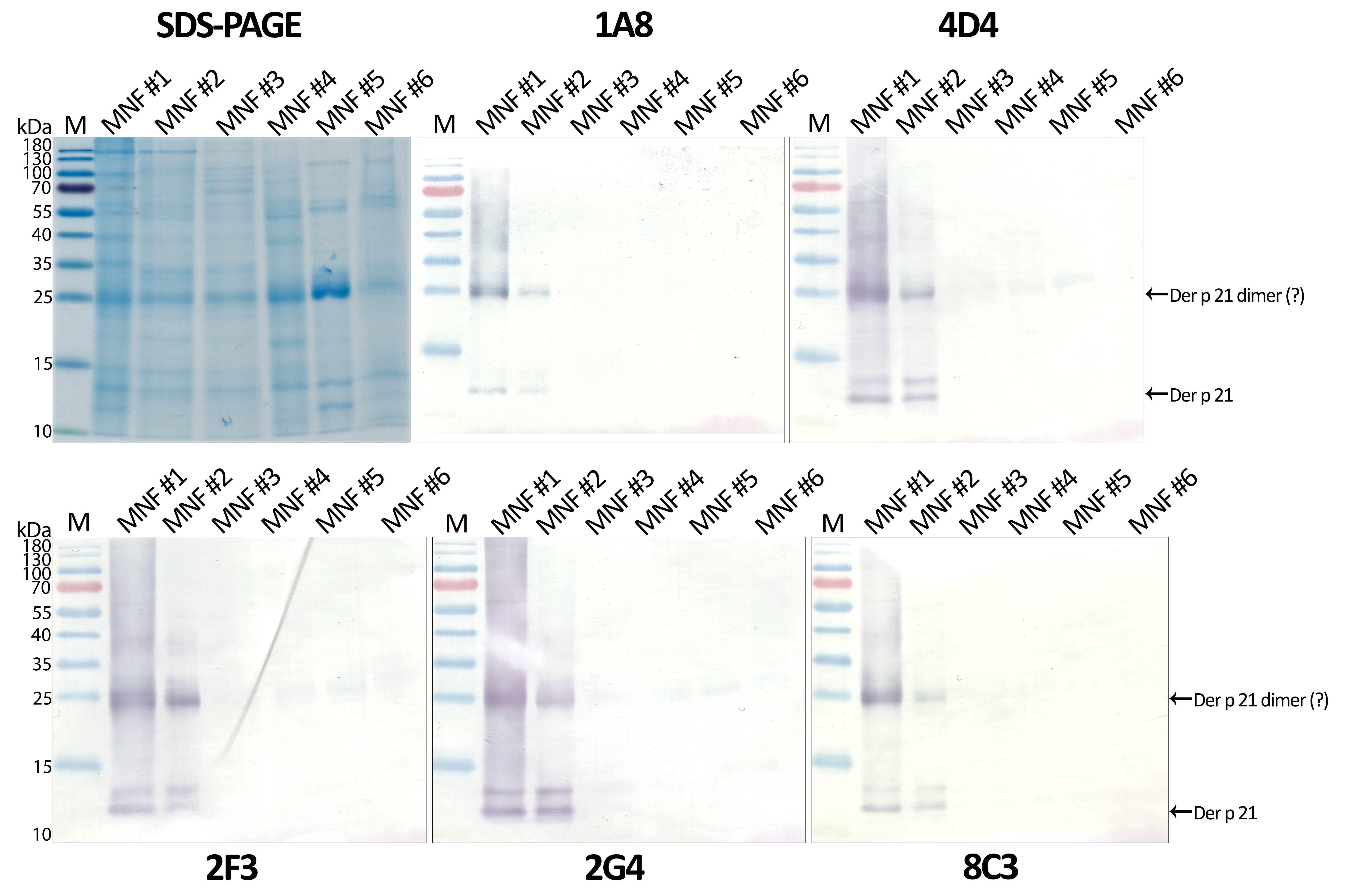

Supplement: Supplemental Information 4 [file peerj-12-17233-s004.png]

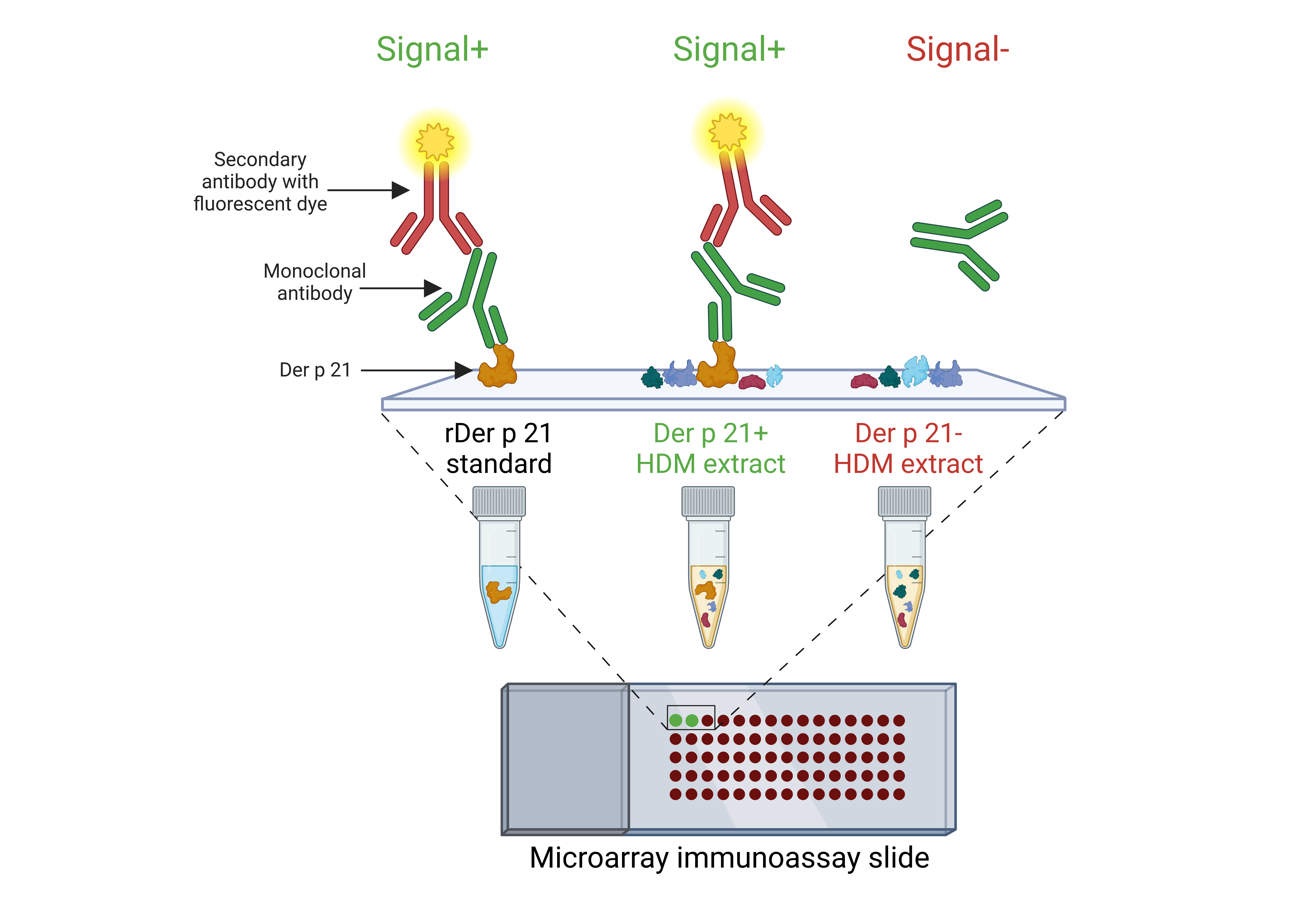

Supplement: Supplemental Information 5 — Created with BioRender.com [file peerj-12-17233-s005.png]

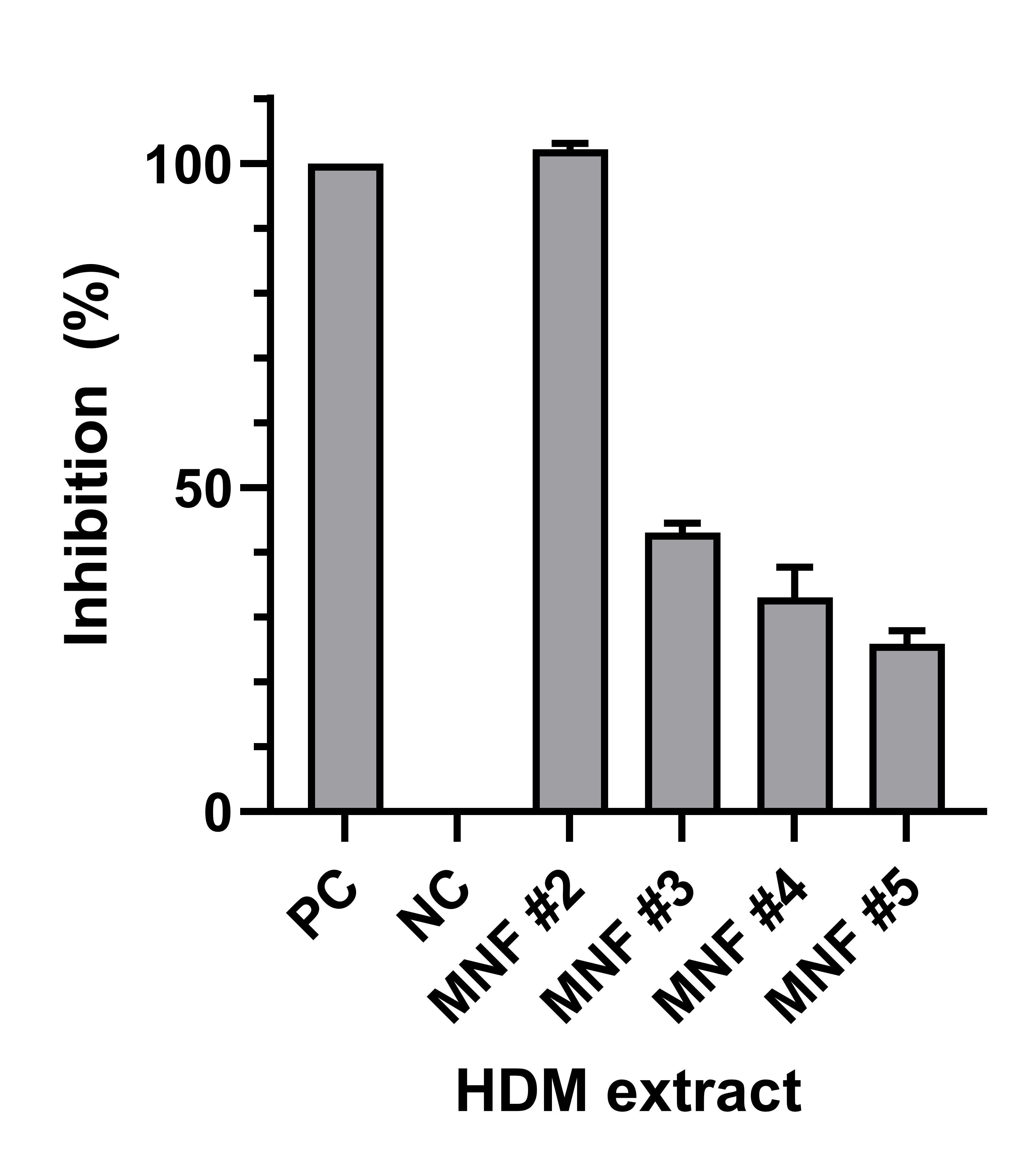

Supplement: Supplemental Information 6 — PC, positive control, serum sample preincubated with purified rDer p 21; NC, negative control, serum sample preincubated without HDM extract. n = 3, mean ± SD. [file peerj-12-17233-s006.png]

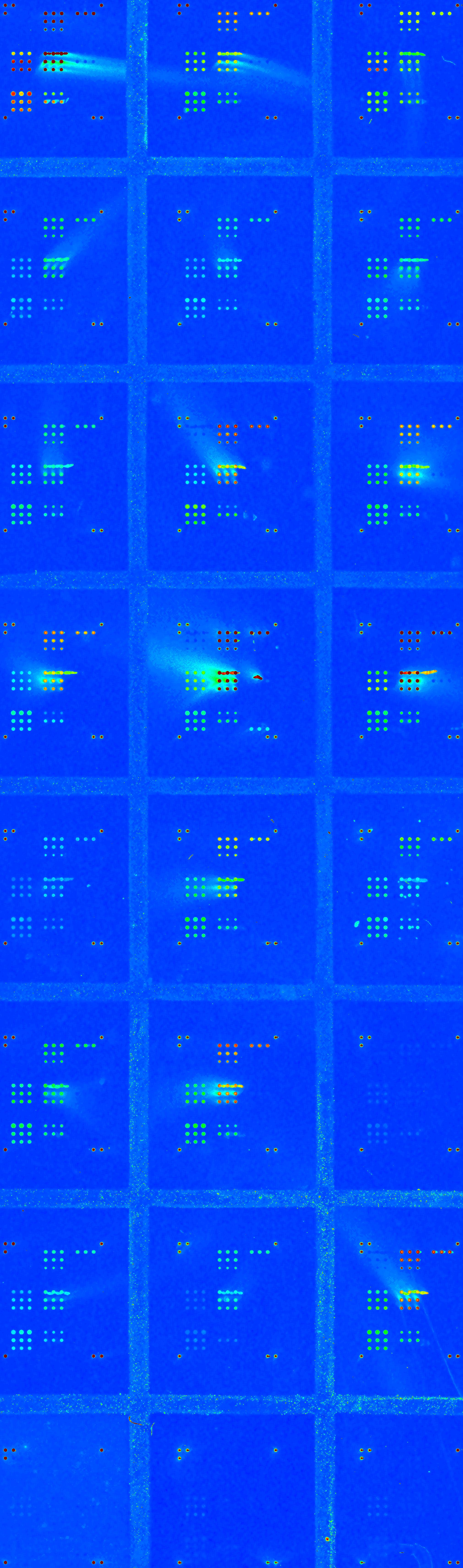

Supplement: Supplemental Information 7 — Scanned image of an allergen microarray glass slide incubated with serum samples of HDM allergic patients. [file peerj-12-17233-s007.tiff]

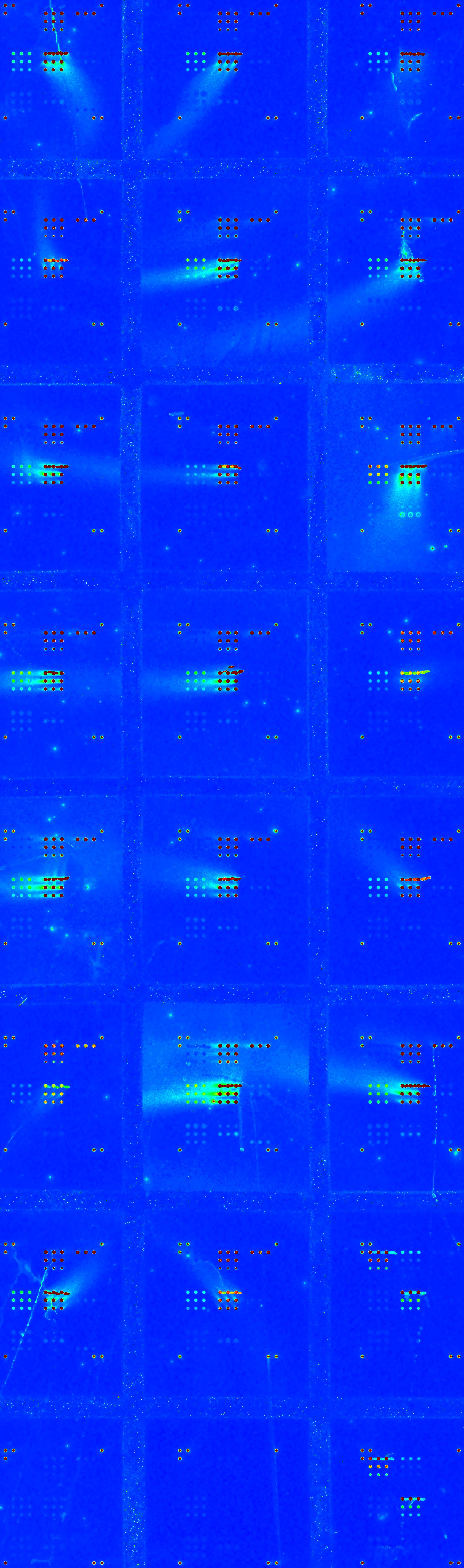

Supplement: Supplemental Information 8 — Scanned image of an allergen microarray glass slide incubated with the MAbs. [file peerj-12-17233-s008.tiff]

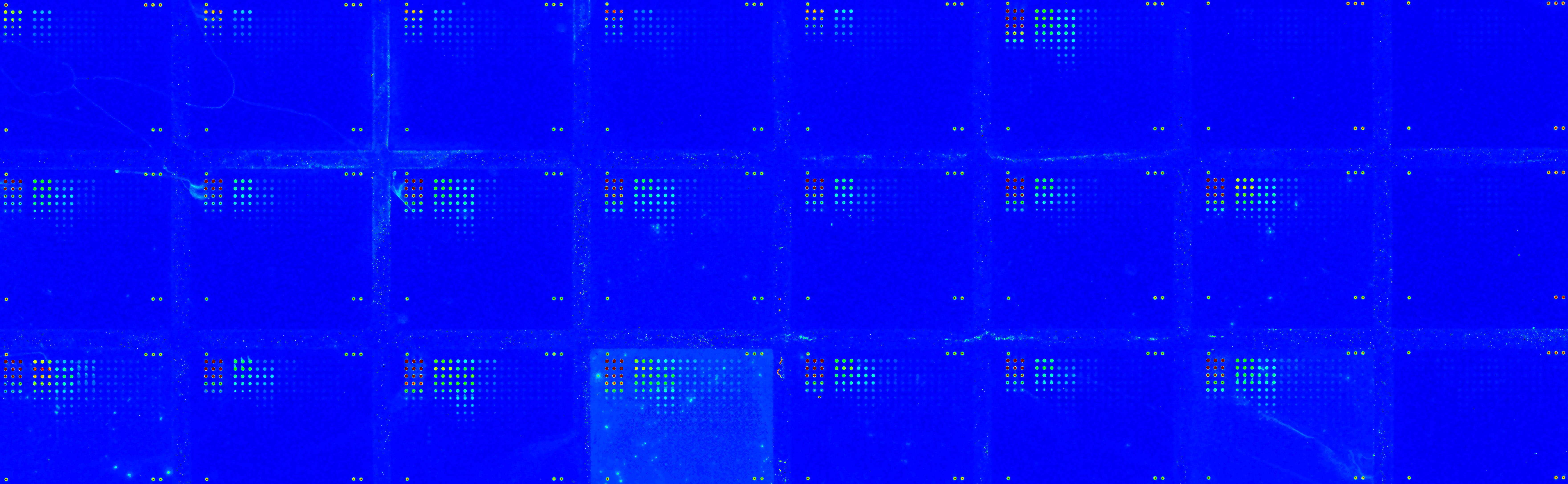

Supplement: Supplemental Information 9 — Scanned image of an allergen microarray glass slide incubated with the MAbs. [file peerj-12-17233-s009.tiff]
