# Supplementary material for: Novel monoclonal antibodies against house dust mite allergen Der p 21 and their application to analyze allergen extracts
Source: PeerJ. 2024 Apr 18;12:e17233. doi: 10.7717/peerj.17233 (PMC11032652; doi:10.7717/peerj.17233)
Supplement: Supplemental Information 10 [file peerj-12-17233-s010.docx]

**Miame Checklist**

(Sections in **BOLD** represent what each researcher must record about

every slide and/or experiment)

Part 1 Experiment description

*“The minimal information required in this section includes the type of the experiment (such as normal-versus-diseased comparison, time course, dose response, and so on) and the experimental variables, including parameters or conditions tested (such as time, dose, genetic variation or response to a treatment or compound).”*

*“this section specifies the experimental relationships between the array and sample entities—that is, which samples and which arrays were used in each hybridization assay. Each of these will be assigned unique identifiers that are cross-referenced with the information provided in the following sections.”*

-         **mouse type**

Not relevant for protein-based microarray

-         **experimental variables (runners vs. non-runners, high fat vs. low fat)**

Not relevant for protein-based microarray

-         **n-count**

Not relevant for protein-based microarray

-         **tissues used for slide**

Not relevant for protein-based microarray

-         **mouse age, and other variables (wean weight, pooled samples, etc.)**

Not relevant for protein-based microarray

Part 2 Array design.

*“The aim of this section is to provide a systematic definition of all arrays used in the experiment, including the genes represented and their physical layout on the array.”*

*“The array-type definition includes information common to all arrays of a particular type (such as glass-slide spotted with PCR-amplified cDNA clones) as well as precise descriptions of the physical content of each element (spot or feature). This section consists of three parts: (i) a description of the array as a whole (such as platform type, provider and surface type); (ii) a description of each type of element or spot used (properties that are typically common to many elements, such as 'synthesized oligo-nucleotides' or 'PCR products from cDNA clones'); and (iii) a description of the specific properties of each element, such as the DNA sequence and, possibly, quality-control indicators.”*

-         **Array series**

Custom made allergen arrays by UAB Imunodiagnostika

-         **Deconvoluted spot list with gene names**

Not relevant for protein-based microarray

-         **Array type (mouse, human, cDNA, oligo, number of genes)**

Protein-based microarray

-         **Array size**

7 x 5.6 mm

-         **Slide type (and coating)**

 2D-Epoxy glass slides (PolyAN, kat. No. 104 00 221)

Part 3 Samples

*“The MIAME 'sample' concept represents the biological material (or biomaterial) for which the gene expression profile is being established. This section is divided into three parts which describe the source of the original sample (such as organism taxonomy and cell type) and any biological*in vivo*or*in vitro*treatments applied, the technical extraction of the nucleic acids, and their subsequent labeling.”*

-         **Cy3/Cy5 labels for tissues**

Not relevant for protein-based microarray

-         **Dye swap? Or reference control?**

Not relevant for protein-based microarray

-         **Labelling protocol used**

Not relevant for protein-based microarray

-         **Sample extraction protocol used**

Not relevant for protein-based microarray

-         **Amount of sample labelled**

Not relevant for protein-based microarray

Part 4 Hybridizations

*“This section defines the laboratory conditions under which the hybridizations were carried out. Other than a free-text description of the hybridization protocol, MIAME requires that a number of critical hybridization parameters are explicitly specified: choice of hybridization solution (such as salt and detergent concentrations), nature of the blocking agent, wash procedure, quantity of labeled target used, hybridization time, volume, temperature and descriptions of the hybridization instruments.”*

-         **Hybridization protocol**

Each allergen and control were printed as a single droplet (~450 pL/drop) in three replicates onto 2D-Epoxy glass slides (PolyAn GmbH, Germany) using sciFLEXARRAYER SX microarray printer (SCIENION GmbH, Germany). The slides were blocked with PBS-T containing 2% BSA (blocking buffer) for 30 min at RT. After that, the slides were incubated for 2 h with sera or MAbs (1:4 dilution and 0.2 µg/mL respectively, diluted with blocking buffer, 80 µL/well). Then the slides were incubated for 30 min with the mouse anti-human IgE Alexa Fluor® 647 (SouthernBiotech, USA) or goat anti-mouse IgG Fc Alexa Fluor® 647 (SouthernBiotech, USA) (1 μg/mL, diluted with blocking buffer, 80 μL/well).

-         **ALL modifications and deviations from the protocol**

none

-         **Manual hybridization or automatic chamber?**

manual

-         **Number of slides done at the same time**

1

-         **Hyb time**

Total time of slide hybridization 3 hours

-         **Number of washes**

10 times in total. 5 washes after primary incubation and 5 washes after secondary incubation with detection reagent. Additional final wash of whole slide was done by immersing the slide into wash glass slide jar.

-         **Amount of labelled sample hybridized**

1 μg/mL, diluted with blocking buffer, 80 μL/well

-         **Labelling efficiency**

Not relevant

Part 5 Measurements

*“Image data should be provided as raw scanner image files (such as TIFF), accompanied by scanning information that includes relevant scan parameters and laboratory protocols.”*

-         **Which version of scanner software used**

MAPIX software 9.1.0 (Innopsys, France)

-         **Laser power for scan**

LaserPower=5.0 W

-         **Instrument model numbers**

InnoScan 710 AL microarray scanner (Innopsys, France)

-         **Must save original .tiff format images (composite image is optional)**

“1 serum.tiff”, “2 specificity.tiff”, ”3 reactivity.tiff”

*For each experimental image, a microarray quantification matrix contains the complete image analysis output as directly generated by the image analysis software (normally provided as separate spreadsheet-type files). Note that for a given image this is a 2D matrix, where array elements (spots or features) constitute one dimension and quantification types (such as mean and median intensity, mean or median background intensity) are the second dimension.*

-         **Normalization protocol**

Not carried out

-         **Does the scanner software subtract background? How much?**

It does not subtract background

-         **Spot raw values, background intensity, ch1 and 2 intensity, etc.**

F635 Median was used as primary raw values for further calculations

-         **Corresponding gene name**

Not relevant for protein-based microarray

-         **Methods of analysis (MAN, Spotfire, Genespring) be detailed.**

*Dose response relationship analysis*

-         **Normalized to controls? Controls removed? All normalization parameters**

*Control spot values were used as reference in comparison with experimental spot values*

-         **Name of Images, Experiment, and location of files.**

“1 serum.tiff” reactivity with serum samples, “2 specificity.tiff” specificity analysis, ”3 reactivity.tiff” reactivity analysis

-         **Lowess or other normalization if used (and parameters)**

 No normalization procedures were carried out

*Finally, the gene expression matrix (summarized information) consists of sets of gene expression levels for each sample. If microarray quantification matrices can be considered spot/image centric, then the gene expression matrix is gene/sample centric. At this point, the expression values may have been normalized, consolidated and transformed in any number of ways by the submitter in order to present the data in a form amenable to scientific analysis. Rather than attempting to impose a standard for gene expression values, MIAME indicates preferred detailed specifications of all numerical calculations applied to unprocessed quantifications in (b) that have led to the data in (c). Experimenters are encouraged, though not required, to provide reliability indicators (such as s.d.) for each data point.*

-         **Output file**

*After scanning the .txt files were generated as raw data files for further calculations*

-         **Normalized ratios**

none

-         **Numerical manipulations**

Each triplicate spot F365 median values were averaged and standard deviation was calculated using Microsoft excel.

-         **Cut off values**

none

Part 6 Normalization controls

*“A typical microarray experiment involves a number of hybridization assays in which the data from multiple samples are analyzed to identify relative changes in expression levels, identify differentially expressed genes and, in many cases, discover classes of genes or samples having similar patterns of expression.”*

-         **Hypothesis**

Binding of the allergen Der p 21, immobilized on the microarray, to the newly developed allergen-specific monoclonal antibodies and IgE from house dust mite allergic patients’ serum samples.

-         **Gene expression patterns found**

Not relevant for protein-based microarray

-         **Controls used, normalization methods used (see above)**

Negative control - recombinant maltose-binding protein (MBP), horseradish peroxidase (HRP), human serum albumin (HSA), phosphate buffer saline (PBS).
